# Observations on Mr. Cribb's Singular Case of Delivery

**Published:** 1805-08-01

**Authors:** 

**Affiliations:** Surgeon, *Sunderland*


### Observations on Mr. Cribb's Singular Case of Delivery; *communicated by* Mr. Dawson, Surgeon, *Sunderland.*

CONJECTURAL remarks, offered in a manly and modest way, on seemingly anomalous cases, are, in my opinion, ever laudable, provided the writer does "not extenuate, or set down aught in malice," and bears in mind the following line:

Vox veritatis, ad cælum volat.

They have the effect of producing discussion; may do good; and if properly conducted, can never do harm. Mr. Cribb's singular case of delivery will be the subject of a few remarks. I think it may be easily simplified, notwithstanding its seeming singularity.

I proceed to do this confidently, and without any apology; I believe none to be required; I am actuated by the love of science, and by no wish to cavil, or hurt the feelings of any man or men.

To me, Mr. Cribb's case appears founded on misapprehension; a misapprehension, which, under similar circumstances, many men might have fallen into. I thus simplify this "singular case of delivery." It was neither an imperforated, nor an united state of the sides of the vagina, but merely an unnatural thickening, and resisting state of the membranes investing the fœtus! This may seem a bold assertion; let me shew it to be almost incontrovertible by attending circumstances. Mr. Cribb says, "On examining, I found a large and hard tumor pressing very low down, and covered by an unusual substance, which felt soft and uneven to the touch."

The hard tumor was doubtless the child's head; the unusual substance, unquestionably, the membranes covering it. Again, "by the yielding of the bones to a moderately firm pressure, there was but little doubt of its being the head of a child; and I could also feel the sutures, though but indistinctly." This is a still more convincing proof, that the "unusual substance, which felt soft and uneven to the touch," was the investing membranes. Had it been, according to Mr. Cribb's opinion, an imperforated vagina, or more correctly speaking, had the surfaces of the vagina been united in consequence of ulceration, and subsequent granulations, he could hardly have felt the sutures of the cranium, as in all human probability there would at least have been half an inch in length of the sides of the vagina united.

united. And again, "neither the *os uteri,* nor *the arch of the pubis were* to be felt." This further corroborates the idea I have ventured to form; the head had passed into the brim of the pelvis covered by the membranes, and completely wedged up the vagina; hence the hand could not pass; and this circumstance fully accounts for Mr. C's finger passing "*readily* into the rectum in every trial."

After this supposed union of the vagina, in consequence of a former parturition, the patient menstruated, and was afterwards impregnated. Now we know very well, a female could not menstruate with an impervious vagina! And we have not now to learn, that a woman cannot be impregnated without semen reaching the vagina, or the uterus! If this female had an impervious vagina, how did she menstruate? and how, which is still more remarkable, did she conceive? The latter circumstance could never take place in a woman with an impervious vagina! The mother thought her "daughter just as *she* was." The woman herself seemed to have no suspicion to the contrary. She had a husband too; did he find any obstacle to coition? This is not mentioned. The probability is, he did not. The fact appears to me to be as follows: The woman in no respect differed from any other, but in a *surprisingly thickened, unnatural, and resisting state of the membranes.* Mr. Cooper had not examined *per vaginam*; at least, he did so under a full conviction of the vagina being impervious. This fact is too notorious to be questioned, as, during the introduction of a catheter, he fancied the vagina to be impervious, and afterwards would not give credit to the pregnancy of the patient. Still the membranes resisted; still pain forced the head of the child downwards into the brim of the pelvis. At this time Mr. Cribb examined, felt the head of the child and the sutures indistinctly, by moderately pressing "an unusual substance, which felt soft and uneven to the touch," the investing membranes. This toughness of the membrane, though rare, is possible. I have heard of some who, at times, used an instrument for the purpose of perforating them.

If what I have stated should, to a thinking mind, appear doubtful, I beg leave to adduce other circumstances to prove the probability of my positions. In the case in question, did the liquor amnii ever escape? Could Mr. Cooper ascertain the rupture of the membranes? Clearly not! If the liquor amnii was known to escape, or the membranes to be perforated, by this gentleman, then the idea of an impervious vagina falls to the ground. For, how how could fluid pass out of a tube that was impervious? And if the liquor amnii did not escape, and the membranes were not ruptured, does not this afford a striking proof of what I have asserted? Mark the consequence; the membranes were perforated with a bistoury, and in due time the woman was delivered! She died too; this was no uncommon event; the consequence of constitutional irritation produced by a violent and long continued labour! I request these remarks to be considered by others as merely conjectural; in my mind, however, they bear great plausibility. Did I think the perusal of this paper would offend any of the ingenious gentlemen concerned in the case, I would commit it to the flames; but I conceive this to be impossible. I have advanced nothing theoretical; I have carefully reasoned on facts related by the Reporter of the case. The opinions I have formed of the case, I purpose to maintain. If any gentleman will convince me I am wrong, I will most willingly relinquish it. Here mere *ipse dixit* will do nothing; we must have strong and sterling facts. The man who convinces me I am wrong, must first prove to me, beyond a doubt, how a woman could menstruate, and conceive, with an impervious vagina! The gentleman who does this, will receive my sincere thanks.

*July* 6, 1805.

